# CineMol: a programmatically accessible direct-to-SVG 3D small molecule drawer

**DOI:** 10.1186/s13321-024-00851-y

**Published:** 2024-05-23

**Authors:** David Meijer, Marnix H. Medema, Justin J. J. van der Hooft

**Affiliations:** Bioinformatics Group, Wageningen University & Research, Wageningen, the Netherlands

**Keywords:** Scalable vector graphics, Three-dimensional structure, Molecular drawing, Visualization

## Abstract

Effective visualization of small molecules is paramount in conveying concepts and results in cheminformatics. Scalable vector graphics (SVG) are preferred for creating such visualizations, as SVGs can be easily altered in post-production and exported to other formats. A wide spectrum of software applications already exist that can visualize molecules, and customize these visualizations, in many ways. However, software packages that can output projected 3D models onto a 2D canvas directly as SVG, while being programmatically accessible from Python, are lacking. Here, we introduce CineMol, which can draw vectorized approximations of three-dimensional small molecule models in seconds, without triangulation or ray tracing, resulting in files of around 50–300 kilobytes per molecule model for compounds with up to 45 heavy atoms. The SVGs outputted by CineMol can be readily modified in popular vector graphics editing software applications. CineMol is written in Python and can be incorporated into any existing Python cheminformatics workflow, as it only depends on native Python libraries. CineMol also provides programmatic access to all its internal states, allowing for per-atom and per-bond-based customization. CineMol’s capacity to programmatically create molecular visualizations suitable for post-production offers researchers and scientists a powerful tool for enhancing the clarity and visual impact of their scientific presentations and publications in cheminformatics, metabolomics, and related scientific disciplines.

**Scientific contribution**

We introduce CineMol, a Python-based tool that provides a valuable solution for cheminformatics researchers by enabling the direct generation of high-quality approximations of two-dimensional SVG visualizations from three-dimensional small molecule models, all within a programmable Python framework. CineMol offers a unique combination of speed, efficiency, and accessibility, making it an indispensable tool for researchers in cheminformatics, especially when working with SVG visualizations.

## Introduction

Cheminformatics knowledge transfer primarily occurs through presentations, published articles, tutorials, and textbooks. In these contexts, three-dimensional molecular visualizations of small molecules play a crucial role in facilitating the understanding of underlying concepts and research outcomes while also adding layers of informative value. To illustrate this point, consider the scenario where a methodology for generating molecular conformations is presented, and its fidelity is assessed by comparing the root-mean-squared deviation of the atom positions to a validated experimental target. In this case, the inclusion of visual representations displaying both the generated conformation and the target conformation serves as an immediate visual indicator of the quality of the generated structure and allows for a visual aid in the quality assessment. Another instance is the portrayal of potential orientations of ligands within a binding pocket of a protein and their spatial proximity to crucial active site residues. A third example is that a researcher might want to demonstrate how specific functional groups within a molecule exhibit closer spatial proximity under specific environmental conditions than would be inferred solely from their skeletal structural formula. In all these instances, graphical representations hold the potential to convey information far more effectively and intuitively than an extensive textual description. Fortunately, a multitude of software applications are readily available to aid scientists and researchers in crafting three-dimensional visualizations of molecules. Among the noteworthy tools in this domain are Jmol [1], 3Dmol.js [2], Blender [3], PyMOL [4], and RDKit [5] each offering distinct capabilities and features for the visualization and analysis of molecular structures.

The way these tools interface with users determines their usability. Standalone desktop applications provide a user-friendly graphical interface for visualizing molecules, but they lack programmatic accessibility, meaning you cannot control them via scripts, and they cannot be integrated directly into other software as libraries. Jmol falls into this category. Web applications are interactive as well but typically render models on the client side, limiting them to JavaScript, which modern browsers support. Examples include 3Dmol.js and JSmol, the JavaScript version of Jmol. Resources such as the Protein Data Bank (https://www.rcsb.org/) and PubChem (https://pubchem.ncbi.nlm.nih.gov/) rely on such tools to display three-dimensional structures. Notably, 3Dmol.js can also be programmatically utilized in Python through an IPython interface called py3Dmol within Jupyter notebooks. Command line interfaces (CLIs) enable users to interact with programs via the command line, although some of these programs may also feature a graphical user interface (GUI). Certain software applications offer multiple interaction methods, such as Blender and PyMOL. Blender and PyMOL are desktop applications that provide both a command line interface and a Python application programming interface (API). Regarding their underlying technology, Jmol relies on a specialized Java-based graphics engine, while 3Dmol.js is a JavaScript library that utilizes WebGL, a JavaScript implementation of the versatile graphics library known as OpenGL [6], for rendering graphics. Blender and PyMOL have their cores developed in C and also employ OpenGL for rendering.

Python is the language of choice for many researchers in cheminformatics as well as other domains of science that deal with molecular information, due to its versatility and extensive libraries. Therefore, it is important to note that the aforementioned tools are not inherently Python-centric. Compiled languages like C and Java often offer faster performance; however, when employed within a Python-first environment, they may introduce additional dependencies, which could be considered excessive when the primary goal is to generate three-dimensional visualizations of chemical compounds. Additionally, users will not have direct access to all the internal states of objects when they can solely interact with the library through an API, although this might be desired by the user when they would like to apply specific stylistic choices in a programmatic way that are not supported by the API directly. RDKit is a widely used cheminformatics toolkit for cheminformaticians working in Python. RDKit can draw three-dimensional conformations of molecular structures and facilitates customization of these visualizations. However, to our knowledge, it is not yet possible to directly output these images as SVGs.

Creating visuals for compounds is typically just the beginning of the process. More often than not, these rendered images find their way into ensemble figures. When creating visuals for this purpose, it is preferred to output them as scalable vector graphics (SVGs). SVGs describe geometries in a vectorized form using an extensible markup language (XML) format, which format is designed to be shareable. This makes SVG easily modifiable either through a text editor or via a GUI within illustration software such as Adobe Illustrator or Inkscape. However, it’s important to note that the graphics rendering engines of the aforementioned molecular visualization tools are not inherently designed for direct SVG output. While plugins such as the render freestyle SVG add-on for Blender [7] or stand-alone tools like gl2ps [8] might extend this capability, creating SVGs from complex three-dimensional models involves a more intricate process.

Complex three-dimensional shapes are often constructed from simpler flat surface geometric shapes, typically triangles. The description of a complex three-dimensional shape involves connecting these triangles through a process called triangulation. The level of detail in the three-dimensional model depends on the number of triangles used. These triangles are then projected into the two-dimensional SVG canvas. However, merely sorting and rendering these two-dimensional shapes is insufficient when two or more model meshes intersect. To address this, algorithms are employed to sort and subdivide the triangles into visible and invisible parts. This recursive computational process is resource intensive. If this process is not conducted with sufficient detail, intersection lines appear jagged, particularly for models with a lot of curved surfaces—like space-filling molecule models depicting overlapping atom spheres. Additionally, when executed at a high level of detail, SVG files may become exceedingly large, reaching sizes in the tens or hundreds of megabytes, even for relatively small models as every individual triangle needs to be defined in the SVG. Another avenue for outputting SVGs is to embed a PNG within an SVG format. This is less desirable because these SVGs do not provide true vector graphics and are no longer editable as SVG files in post-production, thus limiting their versatility.

To tackle these specific challenges, we introduce CineMol—a Python-centric, dependency-free solution for generating true SVG representations of three-dimensional models of small molecules. CineMol employs a straightforward algorithm to rapidly and accurately approximate two-dimensional projections of three-dimensional scenes. It excels in producing compact SVG files, often just hundreds of kilobytes in size, making them highly practical for sharing, drawing molecules, and enabling extensive customization during post-production. CineMol offers users a user-friendly Python library along with a command-line tool, simplifying the process of creating SVG approximations of three-dimensional molecular scenes. Furthermore, we have developed a demo web page where prospective users can effortlessly generate SVG models from SDF files (https://moltools.bioinformatics.nl/cinemol), providing a hands-on experience with our tool’s capabilities.

## Implementation

CineMol approximates the two-dimensional projections of a three-dimensional scene of atoms and bonds on an SVG canvas without computationally expensive meshes or ray tracing. To ensure that CineMol effectively represents intersections while maintaining the high performance that allows it to generate SVGs in a matter of seconds, various methods were employed to limit the number of calculations.

**Fig. 1:**
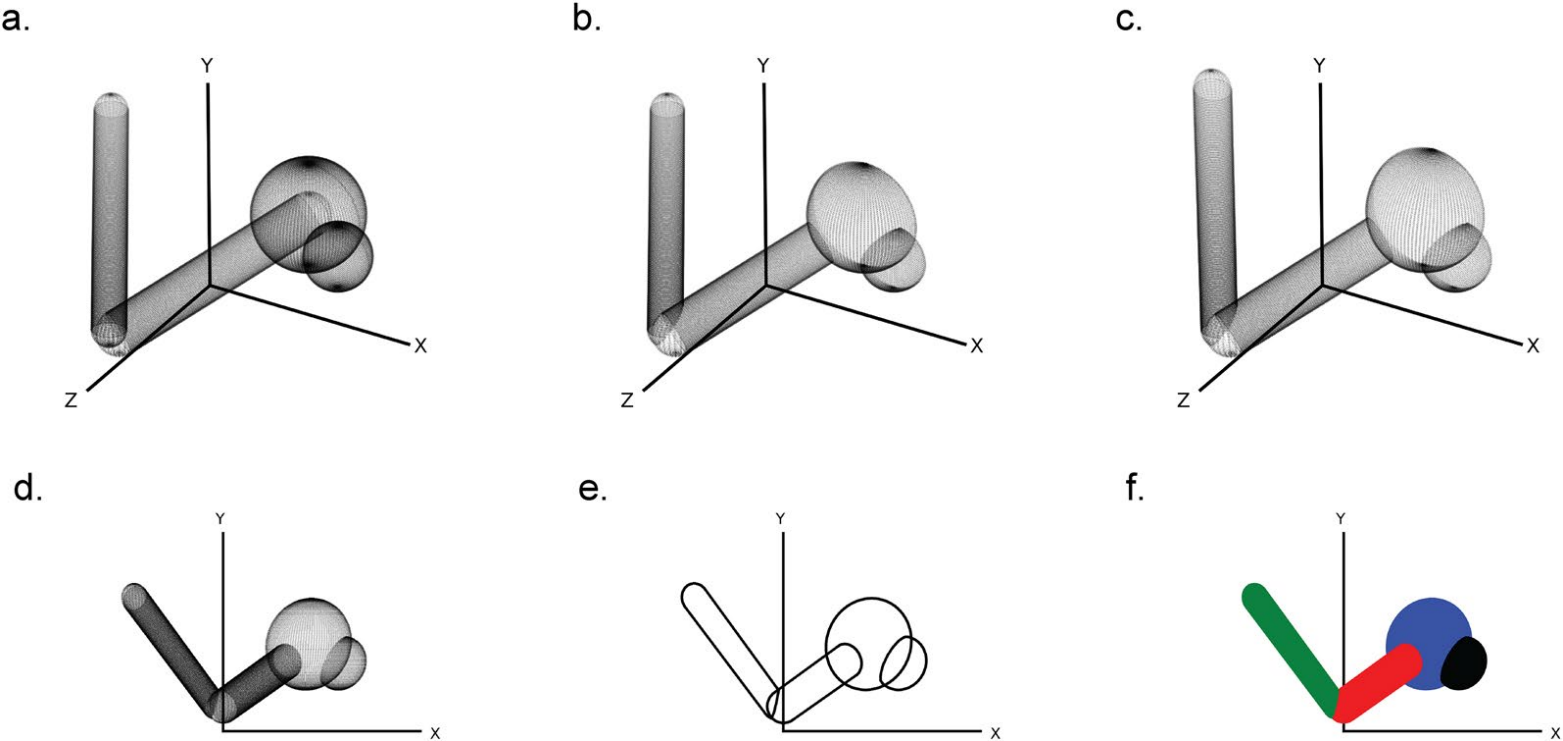
The six steps of CineMol’s algorithm for creating a two-dimensional polygonal projection of a three-dimensional scene. **a** Points are generated on the surfaces of the geometric items in the scene. The number of points generated is based on the *resolution* parameter. **b** The scene items are filtered on the z-coordinate of their centroid and points invisible from the point-of-view of CineMol are discarded. **c** The three-dimensional points are projected based on the focal length of the scene. **d** The z-coordinate is discarded to make the scene two-dimensional. **e** CineMol’s convex hull algorithm calculates the convex shape around each item’s two-dimensional point cloud to create polygonal outlines. **f** Polygons are drawn in their previously sorted order and a fill style is applied. Matplotlib (v3.8.2) was used to create this figure [12]

First, a three-dimensional scene is created by assembling the in CineMol available sphere, cylinder, and wireframe geometries and their styling. This can be done by the user directly when they want full control over the scene and its styling, or by creating *Atom* and *Bond* objects and feeding it into the *draw_molecule* API, together with a global style. The CineMol algorithm will then start by mapping three-dimensional points on the surfaces of the scene items (Fig. 1a). The number of points generated (N) for each geometry is based on the *resolution* parameter.

For a sphere, we generate N points on the surface based on ɸ azimuthal angles (= *resolution* + 1) times θ polar angles (= *resolution* + 1). For a cylinder, we first generate N points (= *resolution* + 1) uniformly distributed points between a start position vector and an end position vector. These points form the centers of circles that outline the body of the cylinder. For every circle, we generate N points (= *resolution*) on the circumference. We cap the cylinder with either no cap or a round cap. The round cap is created by generating points for half a sphere on either end of the cylinder. The *resolution* parameter has no impact on a wireframe geometry.

After generating points on the surface of our three-dimensional scene items, the algorithm filters points based on their visibility from CineMol’s fixed viewpoint (Fig. 1b). CineMol has a fixed view direction towards the origin along the positive z-axis. To speed up the algorithm, we sort scene items from farthest to nearest to the viewpoint based on the z-coordinate of their centroid. As SVG draws polygons in chronological fashion, we only assess intersections for each item between earlier drawn items. For quick intersection checks, we check if the centers of spheres and the central line of cylinders are within each other's vicinity, considering that covalent bonds mainly intersect at their ends with other atoms (i.e., spheres) and bonds (i.e., cylinders). Wireframes are only sorted based on their centroid before drawing. For the remaining points, the algorithm projects their x and y coordinates using a provided focal length (Fig. 1c) and then disposes of the z-coordinate to create a two-dimensional projection (Fig. 1d).

Now, each item comprises a two-dimensional point cloud, representing projected surface points visible from CineMol’s viewpoint, considering that items will be drawn based on their initial z-axis distance to CineMol’s point of view. To establish the smallest encapsulating polygon outlining each point cloud, the quickhull (a two-dimensional algorithm to find a convex hull [9]) is applied (Fig. 1e). The polygons are then drawn from the furthest away to most nearby to CineMol’s point of view, and the supplied fill is applied as styling (Fig. 1f). This fill can be either solid, for a cartoon style, or a gradient for a glossy look (i.e., radial-gradient for spheres and a linear gradient for cylinders). Wireframes can only be styled based on the provided stroke color.

The *draw_molecule* API is wrapped around the *Scene* object and applies a uniform style to all atoms and bonds given to it. The *draw_molecule* API uses the Corey–Pauly–Koltung (CPK) scheme for determining atom and bond colors [10]. The atom radii are sourced from the atomic radii values in the periodic table of elements from PubChem [11]. The *draw_molecule* API is wrapped around a *Scene* object. The *Scene* object is the engine behind the *draw_molecule* API and describes a lower-level abstraction of the scene to draw in terms of sphere, cylinder, and wire geometries. When users would like to fully customize their model instead of using a general style from the *draw_molecule* API they can opt for using the *Scene* object directly and give each geometry their personalized style, if desired.

## Results & discussion

In the following section, we present a series of examples to illustrate the versatility and effectiveness of CineMol, our Python-based three-dimensional molecular visualization tool. These examples serve to showcase the type of depictions and the range of applications that CineMol can accommodate. We will also demonstrate CineMol’s ability to swiftly compute a scene within seconds, showcasing its linear computational scalability, with respect to the number of atoms to draw, when tested on a series of molecular conformers, all while generating compact file sizes. All images in this results section were generated on a MacBook Pro with an Apple M2 chip and 8 GB memory.

### CineMol: a versatile three-dimensional small molecule drawer

**Fig. 2:**
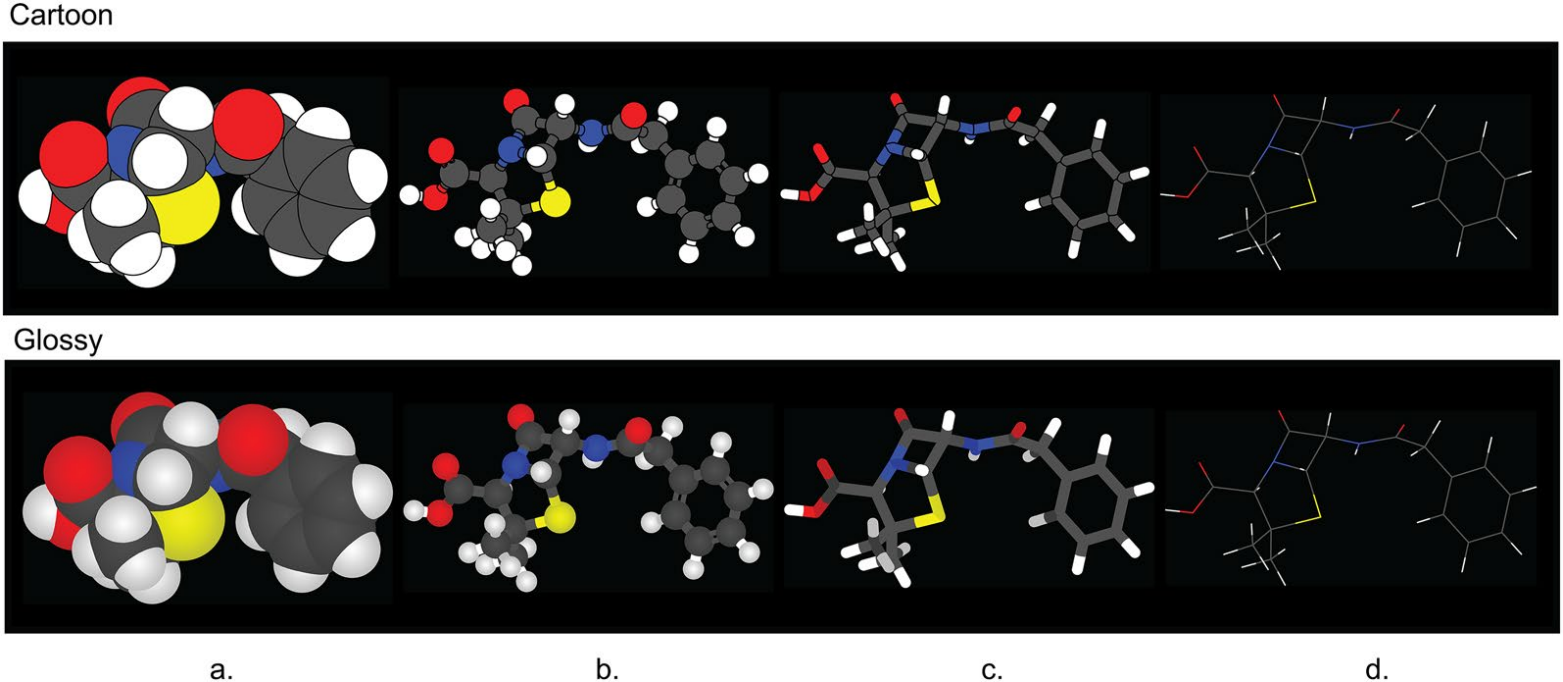
The four depiction types and two fill styles supported by CineMol’s *draw_molecule* API. **a** Space-filling. **b** Ball-and-stick. **c** Tube. **d** Wireframe. The wireframe depiction only supports stroke color and stroke opacity changes and has no separate glossy style. A resolution of 100 and a scale factor of 10.0 was used to generate these images. Algorithm runtimes (cartoon/glossy): space-filling = 1.5 s/1.5 s; ball-and-stick = 1.9 s/1.9 s; tube = 3.4 s/3.4 s; wireframe = 1 ms/1 ms. File sizes (cartoon/glossy): space-filling = 65 kb/74 kb; ball-and-stick: 176 kb/206 kb; tube = 75 kb/93 kb; wireframe = 14 kb/14 kb. The SDF containing the penicillin G conformer used to generate these images was retrieved from PubChem [13]. Adobe Illustrator 2024 was used to compile the SVGs and generate the figure in PNG format

CineMol has a versatile *Scene* object that empowers users to create a wide array of scenes composed of spheres, cylinders, and wireframes, catering to diverse visualization needs. In addition to this, CineMol’s *draw_molecule* API (wrapped around the *Scene* object) provides a user-friendly solution right out of the box, offering four distinct depiction types: space-filling (Fig. 2a), ball-and-stick (Fig. 2b), tube (Fig. 2c), and wireframe (Fig. 2d), along with two unique rendering styles: cartoon (Fig. 2 top row) and glossy (Fig. 2 bottom row). The cartoon rendering style assigns a solid color fill and stroke to each polygon, while the glossy rendering style replicates specular and shadow effects. If the polygons were composed of triangles instead (for example, from the result of triangulation), each surface could have been individually styled to realistically generate specular and shadow effects. Unfortunately, this is not achievable in SVG when the surface is a single polygon.

The *draw_molecule* API has multiple parameters, which are outlined comprehensively in Table 1, providing users with an intuitive and flexible tool for generating molecular visualizations tailored to their specific requirements. Each atom and bond given to the *draw_molecule* API can have its color, radius, and opacity set manually to override the defaults. The *draw_molecule* API can, after installing CineMol with pip, be used directly by importing it in a Python project.

**Table 1:** CineMol’s *draw_molecule* API

Parameter	Type	Description
*atoms*	Atom list	An Atom object takes a unique integer as an index, an atom symbol string, and three-dimensional coordinates of its position (x, y, z). Radius (float), color (RGB tuple of integers), and opacity (float) are optional. This is a required parameter
*bonds*	Bond list	A Bond object takes a start and end index corresponding to atoms in *atoms* and an integer for bond order. Radius (float), color (RGB tuple of integers), and opacity are optional. The bond color is set by the nearest atom by default. This is a required parameter
*style*	Style enum	Either SpaceFilling, BallAndStick, Tube, or Wireframe. This is a requirement parameter
*look*	Look enum	Either Cartoon or Glossy. This is a required parameter
*resolution*	int	Determines the quality of the polygonal approximation of the visible part of every geometric shape. A resolution between 50 and 100 is suggested to be sufficient for most applications. A resolution above 200 only provides marginal improvements. This is a required parameter
*window*	float tuple	Explicitly set the width and height of the SVG. Width and height are not set by default. This is an optional parameter
*view_box*	float tuple	Explicitly set the view box of the SVG. The view box is calculated by the *Scene* object by default. This is an optional parameter
*rotation_over_x_axis*	float	Rotation angle in radians for the model over the x-axis. The rotation over the x-axis is 0.0 by default. This is an optional parameter
*rotation_over_y_axis*	float	Rotation angle in radians for the model over the y-axis. The rotation over the y-axis is 0.0 by default. This is an optional parameter
*rotation_over_z_axis*	float	Rotation angle in radians for the model over the z-axis. The rotation over the z-axis is 0.0 by default. This is an optional parameter
*scale*	float	Scales the coordinates of the model by this ratio. By default, the scale is set to 1.0. This is an optional parameter
*focal_length*	float	A smaller focal length makes the size difference between items nearer to the point-of-view appear larger and the items further away from the point-of-view appear smaller. By default, the focal length is set to None and has no effect on the drawn depiction
*exclude_atoms*	str list	List atoms to filter from the model. This will also filter out the bonds connected to these atoms. By default, no atoms are filtered out
*verbosity*	bool	Set logger level to control verbosity. By default, the logger level is *info*

We have developed two user-friendly interfaces that enable users to swiftly start utilizing CineMol. A GUI built on top of the *draw_molecule* API is available at https://moltools.bioinformatics.nl/cinemol. Additionally, the installation of CineMol ships with a CLI wrapped around the *draw_molecule* API as well, which contains much of the same functionality. Currently, molecules in the form of structure-data format (SDF) files can be supplied to the GUI and CLI. CineMol places its primary emphasis on visualization rather than parsing various file formats. As a deliberate design choice, we have refrained from incorporating third-party libraries for file parsing. This decision allows users the freedom to select their preferred cheminformatics toolkit for this specific purpose, providing flexibility and compatibility with a wide range of data sources.

Furthermore, CineMol does not contain any functionality for generating three-dimensional conformations of compound structures. Consistent with its approach of avoiding dependencies for file parsing, we provide users with the flexibility to select their preferred software for conformer generation, rather than bundling such functionalities. For example, the widely used cheminformatics toolkit RDKit can be used to generate conformers for compounds. This is further highlighted in the section titled “CineMol allows full customization of models for experienced users”.

### CineMol allows full customization of models for experienced users

**Fig. 3:**
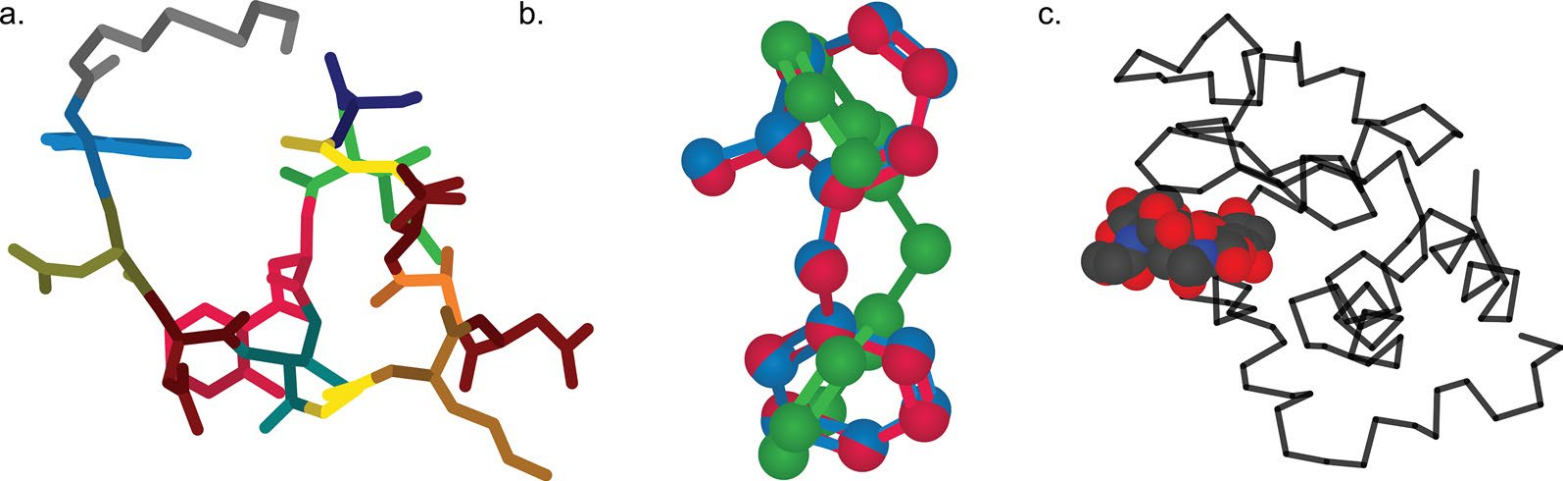
Examples of custom figures created programmatically direct-to-SVG with CineMol. **a** A daptomycin conformer with its monomers highlighted. **b** Three superimposed conformations of o-benzylphenol. **c** The lysozyme 9LYZ with bound bacterial cell wall trisaccharide NAM-NAG-NAM. Algorithm runtimes: 3a = 1.8 s; 3b = 3 min; 3c = 2.3 s. File sizes: 3a = 123 kb; 3b: 211 kb; 3c = 135b. Adobe Illustrator 2024 was used to compile the SVGs and generate the figure in PNG format

Experienced Python users may desire additional customization options for their molecular models, and CineMol offers the flexibility to achieve this. To do so, users can leverage the underlying *Scene* object and supply it with model nodes. These nodes come in three distinct shapes: spherical, cylindrical, or wire, allowing for tailored molecular representations. Each model node is accompanied by its styling. Three examples are shown in Fig. 3, and the exact implementations of these examples can be found in the CineMol GitHub at https://github.com/moltools/CineMol/tree/main/examples.

Figure 3a shows a generated three-dimensional conformation of the non-ribosomal peptide daptomycin that was generated with the wrapper *draw_molecule* API with a resolution of 50. Every different color highlights a distinct monomer in the molecule. The conformation generation and the substructure searches were performed with RDKit v2023.9.6 [5]. Figure 3b shows three RDKit-generated conformations of o-benzylphenol superimposed on each other. To accurately visualize the intersecting spheres and bonds, the resolution was increased to 150 and we instructed the algorithm to not filter nodes for intersecting. By default, the algorithm filters nodes for intersection. This means that a quick check is performed to estimate if two nodes intersect before calculating the exact intersection. Turning off this quick check slows down the calculation but makes sure that every intersection is accurately visualized in this particular case. The *Scene* API allows users to set or include specific calculations to create their own trade-off between accuracy and speed. Figure 3c shows a wireframe of the lysozyme 9LYZ with a space-filling model of the bound bacterial cell wall trisaccharide NAM-NAG-NAM [14]. The opacity of the protein wireframe was set to 0.75, and the model was manually rotated to show the bound ligand more clearly. The PDB file was parsed with the bioinformatics toolkit biopython v1.83 [15].

### CineMol generates SVG drawings with small file sizes in a matter of seconds

**Fig. 4:**
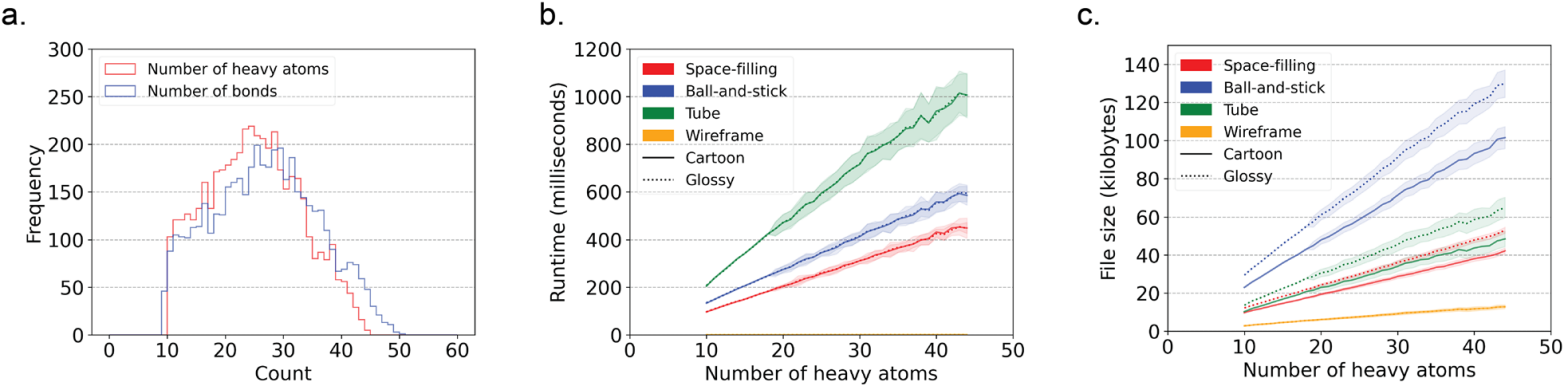
Runtime and file size performance metrics for generating SVG models for 4548 protein-bound ligands from the Platinum dataset. **a** Number of heavy atoms and bonds per ligand in the Platinum dataset. wireframe model SVG files magnitudes smaller than the SVG files for the other model styles. **b** Runtime performance. **c** File sizes. The fill lines indicate one standard deviation. Matplotlib (v3.8.2) was used to create this figure [12]

Figure 4 illustrates the runtime performance and resulting file sizes when generating models for 4548 protein-bound ligand conformations from the Platinum dataset v2017_01 [16]. These results were obtained using the *draw_molecule* API with a resolution of 50 and excluding hydrogen atoms.

Several key observations can be made from the data. First, all atoms in the dataset contain between 10 and 45 heavy atoms and between 10 and 50 bonds, as depicted in Fig. 4a. This demonstrates the applicability of CineMol for molecules of these sizes. It is important to note that a resolution of 50 was used and only heavy atoms were considered for this analysis.

The data reveals general trends among space-filling, tube, and ball-and-stick models. Runtime and file size show a linear relationship (as denoted by Fig. 4b). Tube models exhibit double the runtime of space-filling and ball-and-stick models due to the computationally intensive nature of calculating cylinder-cylinder intersections, since cylinders tend to have more generated points than spheres after filtering. The algorithm does not calculate cylinder-cylinder intersections for ball-and-stick models, as they are typically not visible given the smaller radii of bonds compared to atoms in molecular models. If bond radii were larger than atom radii, a tube model would be more appropriate to generate anyway. Users can customize the behavior regarding which geometries require intersection calculations by directly accessing the *Scene* object.

Furthermore, file sizes for ball-and-stick models are approximately twice as large as those for space-filling and tube models (Fig. 4c). This is expected because ball-and-stick models typically entail about twice as many polygons to describe in the SVG file. The runtime difference between glossy and cartoon styles remains minimal since only the fill step (as shown in Fig. 1f) differs and is not computationally intensive. File sizes for SVG models with a glossy style are a factor bigger than SVG models with a cartoon style (Fig. 4c). This can be explained by the fact that gradients, which are used to create the glossy look, take more characters to describe than a solid fill, which is used to create the cartoon look.

Wireframe models present distinct trends compared to other style types. In wireframe models, no intersections are calculated; instead, only the sorting of individual wires is performed. This process is not computationally demanding, resulting in consistently small runtimes (Fig. 4b). Wireframe model SVG files are also magnitudes smaller than the SVG files generated for other model styles. Line segments in wireframe models are defined by start and end positions, while single polygons consist of numerous individual line segments (Fig. 4c).

In summary, for this set of ligands, the runtime ranges from 1 to 1200 ms, indicating that CineMol can efficiently render similarly sized ligands in seconds with a reasonable resolution of 50. The runtime of CineMol typically scales quadratically with the resolution. For example, drawing a space-filling model of penicillin G, which has 23 heavy atoms, without hydrogen atoms, takes approximately 200 ms for a resolution of 50, approximately 800 ms for a resolution of 100, and approximately 3.2 s for a resolution of 200. A resolution of 100 is sufficient for most applications. Any resolution higher than 200 tends not to lead to visible improvements for most applications. File sizes remain in the range of tens to hundreds of kilobytes across all style combinations.

## Conclusions

In conclusion, CineMol addresses a specific need in cheminformatics by providing a Python-first software package capable of producing precise SVG representations of small molecule models. This tool facilitates enhanced visualization options for three-dimensional molecular structures with a focus on customization and the ability for post-production editing. CineMol’s efficient performance and accessibility make it a valuable tool for researchers and scientists in the field of chemistry and beyond.

## Data Availability

CineMol v1.0.0 is available for Python versions 3.10 and up, has no third-party dependencies, and is released to PyPI (https://pypi.org/project/cinemol/). The source code of CineMol is freely available on GitHub at https://github.com/moltools/cinemol under the MIT license, together with the code to generate any figure present in this article. We have followed the reproducibility and reusability guidelines as described by Hoyt et al. [17], using the *cookiecutter-snekpack* template (https://github.com/cthoyt/cookiecutter-snekpack). A web-based demo version of CineMol is available at https://moltools.bioinformatics.nl/cinemol. We have archived the version of CineMol’s repository used to generate results for this publication with Zenodo (10.5281/zenodo.11242217).
